# Supplementary material for: Comparing autoregulatory progressive resistance exercise and velocity-based resistance training on jump performance in college badminton athletes
Source: PeerJ. 2023 Aug 7;11:e15877. doi: 10.7717/peerj.15877 (PMC10414022; doi:10.7717/peerj.15877)
Supplement: Supplemental Information 1 [file peerj-11-15877-s001.docx]

Effects of progressive versus velocity-based regulating resistance training on the explosive power of the lower limbs in badminton players

Experimental program

1. Declaration of Integrity

This study guarantees the authenticity of the operation in strict accordance with the test protocol and data recording; there is no conflict of interest.

2. Study title

Acute study of incremental and speed-based conditioning resistance training on explosive power of lower limbs in badminton players

3. Source of funding

Self-funding

4. Timeline for the implementation of the research

2023.04.30-2023.05.30 Testing and intervention phase and data collection

2023.06.01-2023.12.30 Data collection, statistical analysis, and writing a paper for publication.

5. Background of the study

Badminton is a complex, fast, dynamic netball sport with specific physical and physiological attributes, court performance, and technical and tactical requirements. Badminton emphasizes a variety of short and explosive movements such as quick changes of direction, jumps, lunges in front of the net, and fast arms. Among them, jumping ability is a key factor in determining the athletic performance of badminton players. Resistance Training (RT) is considered to be an effective method to improve muscle strength, muscle hypertrophy, explosive power, speed and muscular endurance. Strength qualities are known to play a key role in improving and maintaining athletic performance, including increased speed, agility and explosiveness, and even contribute to the development of motor skills [7]. Badminton players often need to use a mid-cycle (2-6 weeks) before a match to convert the daily improved muscle strength into explosive power, laying the foundation for better athletic performance in competition.

In recent years, under the trend of digital and intelligent sports training, Autoregulated Resistance Training (ART) has been proposed and applied to strength training, which mainly monitors and evaluates whether the exercise load is reasonable according to the specific performance of athletes in real time dynamics, and at the same time, by adjusting the training load (including training volume and The main purpose of ART is to monitor and assess whether the exercise load is reasonable based on the athlete's real-time dynamic performance, and to adjust the training load (including training volume and intensity) so that the athlete obtains the appropriate training load for his or her immediate state, thus gaining more effect and reducing fatigue. Among them, Autoregulating Progressive Resistance Exercise (APRE), Rating of Perceived Exertion (RPE) and Velocity-based RPE, VBRT and APRE have been proven to be effective in controlling the intensity of resistance training, and the correlation and difference of the training effects of the three methods have become a hot topic in the current research on autonomic resistance training.

Currently, ART research has focused on the correlation and difference between APRE and traditional Linear Periodization (LP), VBRT and traditional Percentage-Based Resistance Training (PBRT), VBRT and RPE, and different Velocity Loss (VL). Mann's study demonstrated that APRE was better than linear periodization (LP) for strength gain at mid-cycle. Therefore, the purpose of this study is to compare the effects of APRE and VBRT on athletes' jumping ability before competition, which can provide a practical basis for athletes in the competition period to further improve their jumping ability during the mid-cycle.

6. Purpose of the study

Both the APRE and VBRT groups were used as experimental groups and badminton players were used as the study subjects to test the effects of two different autonomic conditioning training protocols on the explosive power of the lower limbs of badminton players.

7. Significance and value of the study

To investigate the study of the effect of APRE and VBRT on the explosive power of the lower limbs of badminton players, to provide evidence for improving athletic performance of the lower limbs of athletes from the practical research basis, and to provide effective empirical reference materials for developing explosive power of the lower limbs of badminton players. From the theoretical basis to provide a basis for enriching the theory of competitive sports training and improving scientific training methods.

8. Research hypothesis and variables

Research hypotheses:

(1) There is a significant improvement effect on the jump height of subjects in the APRE group;

(2) There is a significant improvement effect of RSI and EUR in the subjects of VBRT group.

9. Inclusion and exclusion criteria

Inclusion criteria:

(1) All participants were older than 20 years of age;

(2) At least 3 years of badminton specific experience;

(3) Badminton team of Canton Sports Training Institute

(4) Subjects voluntarily joined the study, signed an informed consent form, complied well and cooperated with the follow-up intervention.

10.Exclusion criteria

(1) Low compliance;

(2) Injury loss or withdrawal;

(3) Failure to cooperate

11. design plan, design model diagram with SPIRIT template

Parallel design, prospective, two-arm randomized controlled trial

12. sample size estimation

Two groups of 21 subjects were recruited, and 18 subjects in the final experimental (n=9) and control (n=9) groups were included in the final statistical analysis.

13. Method of randomization and concealed grouping

Random grouping was performed after baseline testing, using SPSS random numbers to randomly assign participants to two groups: APRE or VBRT. neither subjects nor all investigators could know or decide in advance to which group intervention subjects would be assigned to receive treatment, nor could they infer from the group they were already in which group the next patient would be assigned. Random number table sequential coding was performed for simple randomization grouping, while concealment of the random assignment scheme was performed by a second researcher who generated the random assignment sequence and a third researcher who determined subject eligibility, and by the first author who generated and maintained the random assignment sequence. After grouping, participants were verbally informed of their group assignment by the dedicated coach, and were uniformly advised that no additional resistance training could be performed during the experiment, but normal badminton training could be performed.

14. Measurement indexes

Countermovement jump (CMJ), semi-squat jump (SJ), drop jump (DJ)

15. Definition of the validity of the participants

1. Definition of participant validity

Athletes who have signed an informed consent form, have been screened for entry into the study, are well adhered to, and have completed all intervention training as required by the protocol and process.

2. Definition of Participant Withdrawal

Subjects who signed an informed consent form, were screened and entered the study, and then requested to withdraw before, during, and after the intervention follow-up process.

3. Definition of participant exclusion

Subjects who signed the informed consent form, entered the study but were found to be excluded during project monitoring or at the end of the study because they did not meet the inclusion criteria; did not follow the training requirements for the intervention: poor compliance; did not accept the intervention or did not comply with or assume the responsibilities and obligations corresponding to the informed protocol; violated the study protocol; and withdrew with injury during the intervention.

4. Definition of participant missed visits

Subjects who could not be contacted multiple times or in multiple ways before the end of the project; subjects who did not complete the observation period specified in the protocol.

5. Definition of Participant Mixing

All subjects who participated in additional basketball-specific training were considered;

6. Definition of participant discontinuation

Cases in which the program director or investigator discontinued the subject's continuation of the intervention by combining multiple factors; subjects were found to have poor timing or adverse reactions during the study

7. Definition of participant suspension

A participant who questions the project after completing the informed consent form; a participant who intends to withdraw from the project after completing the informed consent form

16. Definition, identification method and management system of adverse events and reactions

1. Definition of adverse events

Any adverse event that occurs between the time a subject signs the informed consent form and is enrolled in the trial and the end of the post-test, but is not necessarily causally related to the treatment, is considered an adverse event.

2. Definition of an adverse reaction

A reaction that is harmful, not expected, and causally related to the intervention, actually occurring during any of the training sessions during the experiment or during the observation period.

3. Identification method

The occurrence of a harmful, non-desired adverse event that is causally related to the intervention is considered an adverse reaction; those without a causal relationship are considered adverse events.

4. Management system

Adverse events or adverse reactions are recorded faithfully during the trial, including the practice of occurrence, severity, duration, and measures taken for the adverse events or adverse reactions.

17. Recruitment of participants

1. Place of participant recruitment: Digital Physical Fitness Experiment Center of Guangzhou Institute of Physical Education

2. Recruitment methods: Recruitment of participants for the research project through the production of posters, micro-messages and coordination with the head coach of the representative team

3. Screening process:

Athletes who meet the inclusion criteria as initial screening subjects →

No positive cases of physical function movement screening with a score ≥14→

Then strict screening was performed according to the inclusion and exclusion criteria Final screening according to whether or not they decided to resource participation in the study and sign an informed consent form

4. Researchers performing the screening: by third-party personnel independent of this study

18. Collection of general information on participants

1. Researchers who performed the experimental collection: Zijing Huang, Mingyang Zhang and other master students

2. Contents of general information:

(1) Age, height, weight, BMI, injury history, training history, and athlete rank.

(1) Each index of body composition

(2) Baseline indicators and observed performance variables

19. Statistical analysis methods

1. statistical data were analyzed descriptively, Shapiro-Wilk test for normality of the distribution of all variables and Levene test for chi-square of each group was used;

2. independent sample t-test and paired sample t-test were used to analyze differences between groups at baseline and within groups for pre and post-tests, respectively, and Mann-Whitney and Wilcoxon rank tests were performed if normality was not met and expressed as [median (interquartile range);

3. the absolute reliability of the retest was assessed using CV and the relative reliability was calculated using a one-way random effects model with 95% confidence interval (CI) intra-group correlation coefficient;

4. two-factor repeated measures ANOVA for between-group differences;

5. objective analysis based on magnitude inference combined with null hypothesis p-values

20. participant management system

1. written informed consent must be signed by all subjects prior to the intervention;

2. screening of subjects based on inclusion and exclusion criteria for enrollment

3. attention to blinded handling and protection of participant privacy and safety

21. specimen management system

No specimens were collected from the subjects in this study.

22. Drug and equipment management system

No drug interventions were performed in this study.

23. Data management system

All data information was backed up in real time and recorded in the background and entered into separate forms for unified entry and analysis by a third party, while the database was managed jointly by Zijing Huang and Lunxin Chen.

24. Composition and job responsibilities of the Data Security and Monitoring Committee

The data are managed by a third party not involved in the follow-up study and monitored by the ethics committee.

25. Research team

It consists of the study leader, participating researchers, research article writers, test evaluators, data collection administrators, data statistical analysis professionals, and study data checkers.

26. Intellectual Property Rights

All intellectual property rights of the study are vested in Guangzhou Institute of Sports, and the researchers will be attributed according to the size of their contribution to the study.

30. Publication Plan

One paper is expected to be submitted for publication in December 2022.

31. Raw data sharing plan

The data will be made public in the research platform of Guangzhou Institute of Sports within 1 year after the completion of the experiment.

32. Treatment and management of the participants after the end of the trial

After the completion of the experiment, the subjects will be provided with physical functional movement correction and field training equipment.
